# Healthy lives and well-being for all at all ages: expanding representations of determinants of health within systems dynamics and integrated assessment models

**DOI:** 10.1186/s42055-023-00064-5

**Published:** 2023-12-05

**Authors:** Eartha Weber, George Downward, Maria G. M. Pinho, Detlef P. Van Vuuren

**Affiliations:** 1 Copernicus Institute of Sustainable Development, Utrecht University, Utrecht, The Netherlands; 2 Department Population Health Sciences, Julius Center for Health Sciences and Primary Care, University Medical Center, Utrecht, The Netherlands; 3 Institute for Risk Assessment Sciences, Division of Environmental Epidemiology, Utrecht University, Utrecht, Netherlands; 4 PBL Netherlands Environmental Assessment Agency, The Hague, The Netherlands

## Abstract

Integrated Assessment Models (IAMs) and System Dynamic Models (SDMs) are starting to incorporate representations of the impact of environmental changes on health and socio-economic development into their modelling frameworks. We use this brief review to provide an overview of how health and well-being are currently represented in IAMs and SDMs. A grey literature search on 12 selected model host websites and their corresponding Wiki pages was conducted. Model descriptions, coverage and publications were then tabulated. Additional potential determinants related to health were then suggested based on emerging environmental health literature. Based on these tabulations, it was determined that many individual health outcomes are not represented and thus not analyzed. Social well-being is not represented at all. Additionally, potentially health relevant determinants such as chemical or metal exposure and water pollution are rarely represented in models. Most models have representations of climate, outdoor air pollution and food availability. Air pollution was the most analyzed determinant, especially in relation to its respiratory effects. We suggest that future modelers incorporate more representations of environmental determinants influencing health, and to analyze all available determinants in relation to a wider array of health outcomes. Perhaps, if and when broader determinants of health are represented in IAMs and SDMs, then a composite of these determinants could be used to determine a population’s ability to achieve elements that also contribute to social well-being.

**Supplementary Information:**

The online version contains supplementary material available at 10.1186/s42055-023-00064-5.

## Introduction

In 1948, the WHO defined health as the “state of complete physical, mental and social well-being” [1]. This definition was extended in 1986 to incorporate the understanding of health as a process/resource [2]. Achieving universal health and well-being is an important policy goal in many countries: good health allows for more complete participation in families, communities, workforces, and environmental stewardship to name a few [3–6]. The concept of health expressed as a dynamic interaction between multilevel determinants, emerged as knowledge progressed. Commensurable to these dynamic understandings, the world has developed an agenda through Sustainable Development Goal 3 (SDG 3) that aspires to achieve “healthy lives and well-being for all at all ages” by 2030.

Health status is dependent on a range of factors (physiological risks, exposure to environmental factors, socio-economic status, lifestyle factors, etc.) simultaneously, including the capacity to respond to and maintain health status (such as access to treatment or healthcare). Determinants of population-level health and well-being are, among others, embedded in the environment that populations live in. The determinants manifest in positive or negative health outcomes through a combination of patho-physiological mechanisms interacting with social and multi-level institutional interactions and networks [7, 8]. A recent literature review showed that current projections for diseases as a consequence of environmental change are typically still limited to looking into a few individual diseases and stress factors – and hardly account for socio-economic changes [9]. Thus, conducting a comparison of the models themselves, can allow for identifying which health determinants are covered and which can be potentially added.

Tools are needed that can project whether or not the quantifiable aspects of SDG 3 can be achieved based on various transitions. Integrated models such as Integrated Assessment Models (IAMs) and Systems Dynamics Models (SDMs) could possibly form a basis for the systems perspective of future health. IAMs and SDMs are tools used to understand the interactions between the environment and human systems (usually energy and economic sectors) based on various emission pathways [9–13]. IAMs and SDMs could be useful tools to assess how environmental or human systems impact human health and well-being and vice versa.

In this paper, we focus on the ability of IAMS and SDMs to project the relationship between environmental factors and health. We first provide a review of how selected IAMs and SDMs represent health and potential health-relevant determinants related to the environmental and human systems that they are represented in. Second, we examine the publications that the IAM community has conducted which have already analyzed the impacts of environmental determinants on health outcomes or aggregate measures. Third, we discuss how IAMs and SDMs can be enhanced and a potential method to approach modelling mental and social well-being for the purpose of future projection [12].

## Methods

### Description of current models

For our research, we selected a set of representative models – mostly listed at the Integrated Assessment Modelling Consortium (IAMC) website [13]. Models were selected based on their description on the documentation at the IAMC website [14]. The key criterion used for inclusion was that the model should have a human/earth system coverage and have some publications related to health. The GAINS (Greenhouse Gas Pollution Interactions and Synergies) model was added as it represents a different type of IAM – focusing specifically on air pollution. The first search was conducted in 2021 and then updated in December 2022 (See Supplementary Table 1). Information was collected on the models themselves such as model type and analytical framework. A summary is provided in Supplementary Table 2.

**Fig. 1:**
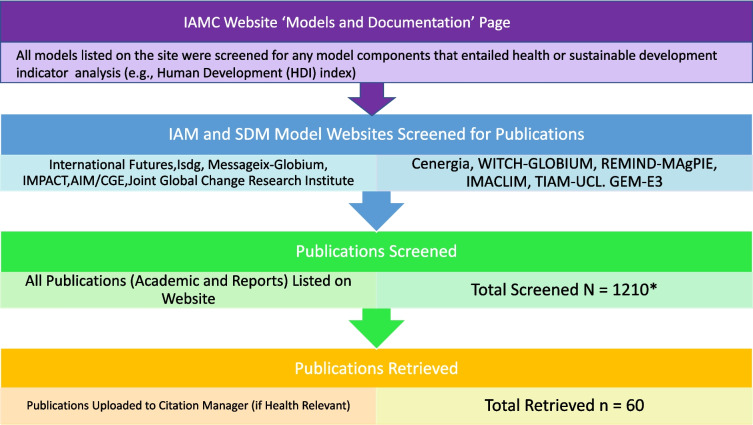
Flowchart summarizing websites analyzed and publications screened

Based on the information provided on the websites we created a table that indicates whether health-relevant parameters are covered for each model. This includes drivers, environmental risk factors known to influence health, and health elements. The purpose was to identify the extent to which the models have or could possibly cover health issues (Fig. 1). We categorized the representation of these parameters into four distinctions: ‘no’,’exogenous’, ‘sometimes’, A ‘yes’ indicates that the element is endogenously included, while “sometimes” indicates that it is present yet rarely or selectively used. In structural equation modelling the distinction between endogenous and exogenous representation is relevant. Exogenous representation represents an external assumption. For instance, population can be exogenously assumed in a model (meaning not determined by other variables) implying that no further dynamics are included. Endogenous representation means a dynamic description in the model itself.

### Examples of IAM health studies

**Fig. 2:**
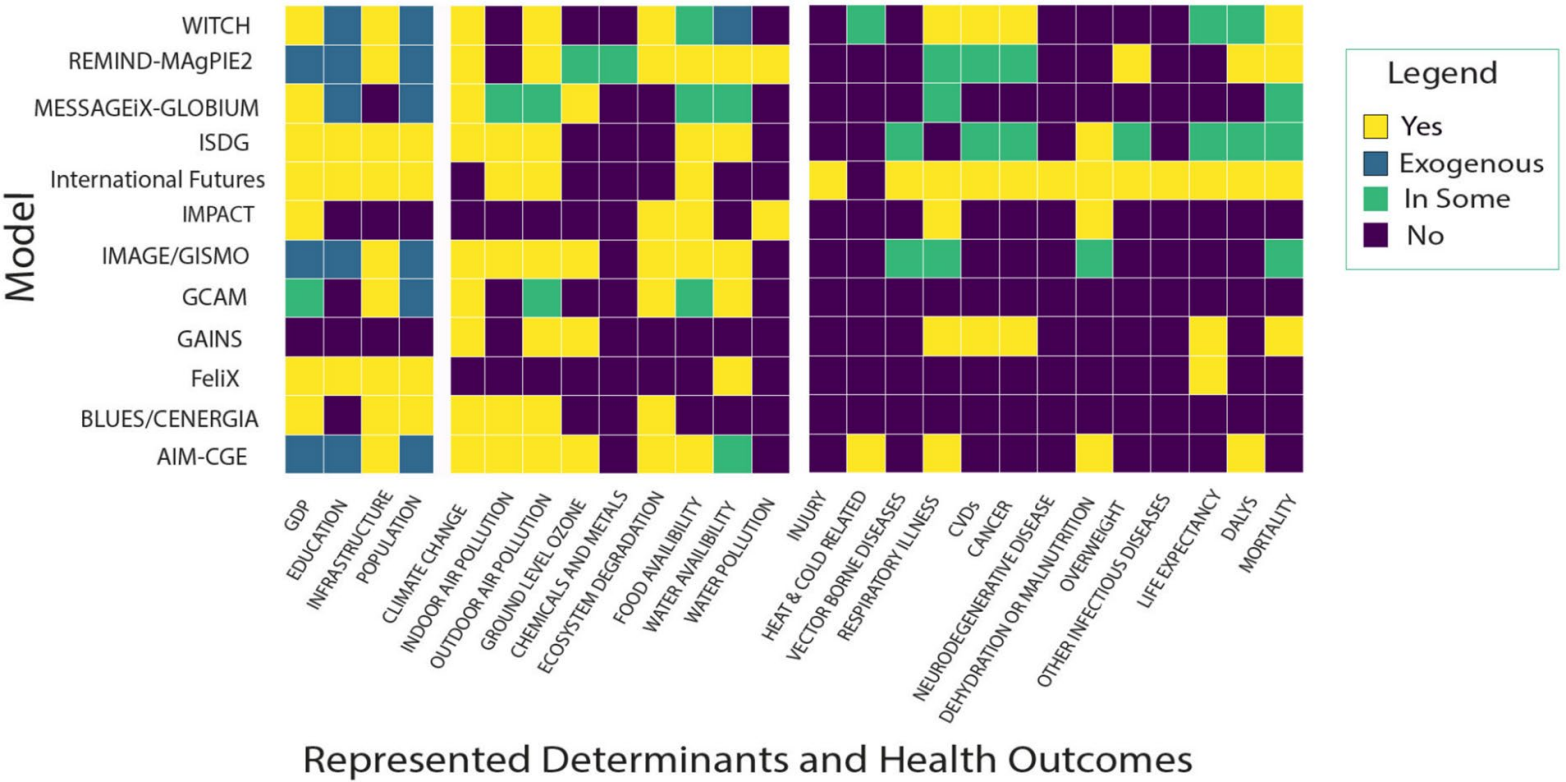
Model coverage of health determinants and health related outcomes

**Fig. 3:**
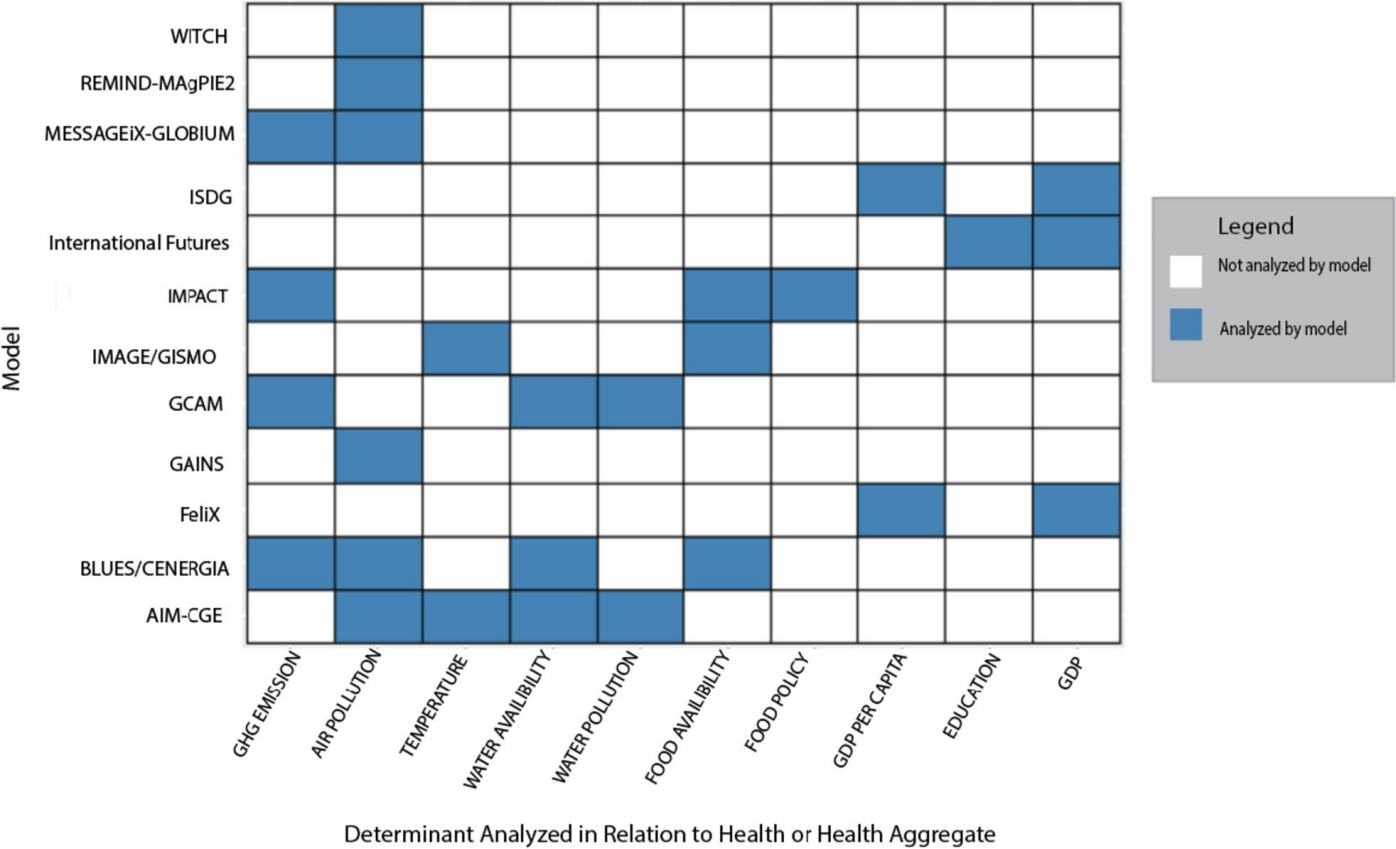
Publications coverage on determinants and health outcome by each modelling community

A second table was made to show what relevant determinants have been modeled in relation to their impact either on health or on the population (e.g. food prices). Short descriptions of the corresponding publications are explained in the results section under ‘Current IAM’s in Relation to Health’. The information collected from grey literature search documentation table template was obtained from the University of Toronto Library and is listed in Supplementary 1 and data extracted from publications is listed in Supplementary Table 3 [15]. Briefly the data extracted and listed in Supplementary Table 3 was the distal or proximal determinant represented, the scenarios used, the health related outcome, the health analysis tool and which references were included Figs. 2 and 3.

### Ways of moving forward

Two plausible ways that IAMs/SDMs can be enhanced are then discussed. The simplest is by incorporating a more diverse set of environmental exposures to risk on health outcome relations. To demonstrate this another tabulation was developed. IAMs/SDMs could enhance or expand current models based on both possible additional risk factors and its relation to health outcomes or aggregate indicators (Supplementary Table 4). Risk factors (additional determinants) were only included if there was a global dataset available with data on the risk factor and these datasets are also listed in Supplementary Table 4. IAMs and SDMs can also be enhanced by not only examining the presence or absence of diseases amongst a population but to also attempting to capture a more holistic understanding of what it means to be healthy. We narratively explore this possibility using Max-Neef and more recent literature emerging from positive psychology [16].

## Results

### Model coverage

#### What are relevant health determinants and variables already represented in IAMs/SDMs?

Figure 1 depicts what socio-economic drivers, health relevant environmental risk factors and related health outcomes or aggregate measures are currently represented in the analyzed IAM and SD models. A causal chain diagram published previously is used to show important known linkages between drivers of environmental effects and health outcomes can be seen in figure two of the referenced paper [17].

#### Socio-economic drivers

Almost all models included socio-economic determinants such as education or population, but in most cases as exogenous assumptions. Models which endogenously represent population changes (such as *International Futures or iSDG*) have the advantage of capturing important feedbacks in terms of demographic changes in sub-populations which are particularly important when analyzing health impacts. Note also that *iSDG* (formerly named *Threshold 21)* is a customizable model that is created for each country of interest based on a participatory process (e.g. in the model developed for Ghana, the prevalence of malaria/AIDs is incorporated as an influencer of total factor productivity on economic product) [18].

#### Environmental determinants

Most models represent environmental health determinants. All models represent climate change, with 10 doing so endogenously. Correspondingly, 10 of the 12 represent outdoor air pollution as these are generated based off an estimation of PM_2.5_ concentrations calculated from climate emissions. Food availability (*n* = 9) was the next most represented environmental determinant, followed by water availability (*n* = 8), ecosystem degradation (*n* = 7), and indoor air pollution (*n* = 6). Chemicals and metals, as well as water pollution were the least represented environmental determinants, being represented in *Remind-Magpie2* and *IMPACT* models respectively.

The degree of representation does vary per model. In *International Futures (Ifs)* and *FeliX*, there is a simplified endogenous representation of climate with some exogenous features. In *IMPACT*, the climate system is exogenous.

#### Health outcomes, proxies or aggregate indicators

Health outcomes are less well represented. Respiratory illnesses and mortality are the most represented health outcomes across all models, being present in 8 models. Injuries, heat or cold related illnesses and infectious diseases were rarely represented (injuries are represented in *International Futures*, heat and cold in *WITCH*, and *AIM-CGE* and infections in *International Futures* models respectively). Four models have representations of dehydration or various forms of malnutrition. *International Futures* and *IMAGE/GISMO* use calories per capita to produce BMI or undernourishment prevalence.

*International Futures* has the most coverage of varying health outcomes within the models we examined. *iSDG* and *FeliX* only model health on the basis of socio-economic drivers (such as GDP or health expenditure) as it relates to changes in health care access or life expectancy. In the case of iSDG, it should be noted that the representation does depend on the country version. IM*AGE* uses the health model *GISMO* but here the calibration is out of date. *Message-IX* emissions are soft-linked to the *GAINs* model, allowing to assess health loss from air pollution. For *WITCH,* the model represents action to reduce health risks from the impact of extreme temperatures or air pollutants through internalization of costs which are determined by an outside model (e.g. by *GAINS* or *FASST®* or statistical emulation for extreme temperature translated into energy demand) [19].

### Current IAM Publications related to health risks

#### What determinants have already been analyzed in relation to health?

Figure 3 presents the health-related studies performed by the model teams. Here, the environmental determinant included by each model team is specified (if an analysis was available, it was labeled as ‘yes’ and indicated in blue). Both distal (socio-economic) and proximal (biological exposures) causes are presented [20]. Air pollution is the most analyzed health risk. In most cases, ambient air pollution was assigned by estimating PM_2.5_ or PM_10_ emissions (or via a GHG proxy) and calculating health impacts using specific models such as GAINS, TM5/FASST or BENMAP-CE. In some cases, a comprehensive health assessment model was used (e.g. *GISMO* in *IMAGE*). Indoor air pollution is analyzed in an entirely different way (by International Futures and IMAGE/GISMO respectively) and based on the relationship between the distal determinants of GDP per capita and education on the distribution of household use of solid-fuels.

Despite climate change being represented in nearly all of the models only 2 (*IMAGE/GISMO* and *AIM-CGE*) analyze the direct impact. IMAGE/GISMO examine temperature and precipitation in relation to malaria suitability, and *AIM-CGE* examines economic losses due to lost working time and shifts in outdoor working time as a potential adaptation strategy [21, 22]. Many models indirectly represent the impact of climate on water (*n* = 8) and food (*n* = 9) availability (often expressed in population suffering from hunger). However, only some teams analyzed the impact of real health parameters. *IMPACT* analyzed how food availability (which can be altered due to climate change) impacts disability adjusted life years (DALYs) and attributable deaths [22–24]. *IMPACT* also examined taxation on red and processed meat and the corresponding reduction in consumption, reporting reduced deaths from non-communicable diseases like heart disease and stroke. *IMAGE/GISMO* analyzed climate impact scenarios in relation to under 5 (U-5) mortality. There is also literature looking into impacts of climate policy as this could directly (via diet choice) or indirectly (via land competition) influence health. Plenty of literature examines calorie availability or hunger but not much links it to specific health outcomes. However, an IMPACT study analyzed how subsidization of fruit and vegetable intake and taxation of red meats can simultaneously reduce GHG emissions and deaths from metabolic disorders, outweighing its risk on food security in the form of undernutrition if policy exceptions are made in food insecure regions [25, 26]. As an example of studies looking at food availability impacts, *BLUES/CENERGIA* examined the perceived competition between use of biomaterials for energy and land use relative to land use needed for agricultural production. Finding that food availability was not threatened in Brazil [27].

Other modelling communities such as International Futures, *ISDG*, and FeliX use a ‘distal-driver’ approach to determine health impacts and generally do not account directly for pollutants or other environmental risks. *International Futures* predicts age and sex mortality from GDP per capita, total years of education, and smoking. The determinants are also used to compute DALYs. To calculate the difference between a ‘proximal exposure’ such as indoor air pollution, an adjustment is made by adding additional factors related to cookstove use relative to no cook stove use based on historical relationships [28]. Similarly, *FeliX* measures an indicator that represents achievement of SDG 3 (good health and well-being) and uses GDP per capita to compute a change in life expectancy based on how education and population growth also changes. i*SDG* customizes its model renditions in collaboration with national governments and links SDG 3 to increased public expenditure both in the healthcare system but also in relation to family planning [29, 30].

### Plausible health and well-being representation based on diverse conceptualizations of environment

#### How can IAMS be further developed when it comes to health modelling?

The overview above shows that the representation of health in IAMs is still, with only a few exceptions, very basic. However, the presence of environmental risk factors in the models (climate, air pollution, water scarcity and malnutrition, water pollution etc.) allows the assessment of health outcomes (morbidity and mortality) via the causal chain. The mortality rate can be modelled by distinguishing components representing the attribution to specific environmental factors and a non-attributable component (representing other causes of death). Incidence and case fatality rates (the ratio of the number of deaths from a specific disease to the number of diagnosed cases) can be based on various health-risk factors. Such models can take the health-risk relationships from the literature and calibrate the overall data of disease/demographic data. Some of the models are already using such approaches either for several determinants (like GISMO) or for only one (air pollution). Therefore, there is ample opportunity to also connect determinants already represented with determinants such as temperature, food, and water. A list of relationships between diverse conceptions of environment an potential constituents of health and well-being that could potentially be modelled can be seen in Supplementary Table 4. Diverse conceptualizations are separated into three and are obtained from biology, public health and environmental sciences [31]. The first from biology are, traditional integrated environmental-exposure response models to risks like pollutants on diseases [32]. Second an idea obtained from public health is materials and the built environment (which are important for shelter, energy access and mobility) as social determinants of health or well-being [33, 34]. The last environment concept comes from environmental sciences and is entitled the ecosystem services approach whereby preserving ecosystems can fulfill human needs such as heat, recreational space and noise reduction [35].

In addition to the exposures already represented in IAMS, others can be represented in relation to health such as nitrates in water, extreme events, and chemical or metal pollutants. Emerging data sets and insights on the impact of light pollution and environmental microbiome could also create more opportunities for connecting novel environmental factors to health within models [36, 37]. Some brief examples include untreated water in its relation to infectious disease, and pharmaceuticals in water and their relationship to endocrine disruption and anti-biotic resistance. If consideration of materials and the built environment are incorporated then shelter can both be a source of protection from the elements and also be designed in a way for urban heat mitigation. Lastly, the influence of land use and bio-diversity can be incorporated by using an eco-system services approach whereby preserving nature can have impacts such as reducing stress, increasing physical activity, reducing noise, increasing pollination, and increasing tourism [31, 35].

### Going beyond the presence or absence of disease: mental and social well-being

So-far, if a health analysis was conducted by the modelling community the focus has primarily been on the biomedical health concept, i.e. the absence of disease. However, as already described, health has a much broader definition. One of the fundamental purposes of ensuring health within development is so that we as humans have the opportunity to lead meaningful and fulfilling lives. The traditional approach in development, thus far has emphasized economic growth as the indicator of human prosperity in terms of being able to achieve fulfillment of minimum basic needs. Models often equate and use GDP per capita as an indicator of welfare. Historically, progress on extreme poverty has been achieved through increased economic growth, however sustainable and inclusive growth has not been prioritized. The richest part of the world consumes and contributes most to global emissions. At the face of it, perpetual growth of material wealth seems at odds with sustainability and some academics argue that there is a ‘trade-off’ between preserving the earth and lifting people out of poverty. Absence of extreme poverty is often directly equated with well-being in modelling communities however extreme materialism does not linearly equate to extreme well-being. Capturing non-material mental and social well-being determinants can help aid in transitioning towards environments and structures that enable and reinforce sustainable practices where overconsumption is rampant.

One approach to do this is using and adapting Max-Neef’s ‘Human Scale Development’ where he postulates that ‘quality of life depends on the possibilities people have to satisfy their fundamental human needs’ [38]. If human needs are understood as a system, then the satisfiers of these needs are ways to achieve them and correspondingly these satisfiers can be modelled. For example, the need of subsistence can be fulfilled by food and shelter [39]. At the time of creation there was not a consensus on whether fundamental human needs existed. Now, there is emerging psychological literature showing that there is some universalism in what needs influence social well-being. Tay et al. have found that around 60% of social well-being can be determined by basic human sustenance needs and safety which tend to be country specific, while three other needs – social belonging, autonomy and mastery share similar orders of importance across countries [12]. Thus, modelling teams can creatively create or use available indicators that capture diverse satisfiers of these needs and correspondingly adjust the importance of the satisfiers in different contexts to capture the well-being component of health [40]. Examples of satisfiers for the needs of social belonging include settings where interaction can occur like schools, parks/recreation spaces etc. for autonomy it can be equal rights, for mastery access to education or vocational job training across the lifespan. By capturing mental and social well-being in models, the broader concept of health can be adequately represented.

## Discussion and conclusion

In this paper we examined the main models and approaches used in the IAM and SDM community in relation to examining health impact. We discovered that many relevant determinants are already covered in the models, however not many assess the determinants in direct relation to health outcomes. Air pollution in relation to health outcomes are assessed the most. Further more sophisticated understandings of health such as mental or social well-being or resilience of geographical areas in response to change are not examined. Once coverage of health outcomes (such as disease and morbidity) are assessed, model teams can further develop by attempting to measure the determinants of mental/social well-being or by incorporating ranges of level of resilience capability in response to changes.

According to the Lancet, environmental contaminants such as air and metal pollutants (e.g. lead, polychlorinated biphenyls, organophosphate pesticides etc.) are responsible for 1 in 6 deaths worldwide and improvements in household air pollution levels have been offset by increased ambient air pollution and chemical pollution [41]. IAMs and SDMs are adept at linking multiple relevant drivers and sectors together. Fundamentally, this can allow for creating and assessing multi-level strategies and policies which can be critical for long term and effective progress across many health outcomes and determinants of psycho-social well-being. Despite the potential of integrated modelling techniques we found that none of the modelling communities analyzed or published on the linkage of all possible environmental health risk combinations on health outcomes let alone combinations of risks.

General critiques of IAMs and SDMs include, the lack of certainty, and an inability for the models to be validated [42, 43]. Validation of IAM models as well as any other models trying to analyze future outcomes is an ongoing concern [44]. Historical analysis can be one way of evaluating the prior robustness of model outputs, but future uncertainties can be difficult to predict [44, 45]. Uncertainty can result from many sources some of which include extreme climate events being unpredictable since the climate is non-linear and because future rapid technological or social change is not easy to predict [45]. The community aims to address this concern through transparent documentation of the structure, code and variables used [13]. Model documentation is done via the harmonized IAM model documentation, while key input and output data is – again in a harmonized form – made available via the IAMC database provided by IIASA [46, 47].

Another source of uncertainty is that in IAMS the mitigation of climate change is often evaluated in a cost–benefit manner. Using solely cost–benefit analysis the monetary value of saving lives and the ecosystem are continually contested by policy makers and economists [43]. We think that by expanding coverage of IAMS by directly providing a number of lives lost or affected by health conditions due to environmental determinants somewhat bypasses this issue since there are numerous scientific studies which confirm that contaminants and pollutants prematurely end lives. Human biology is not likely to change drastically in the next two generations. Thus, coupling potential health impacts to IAMs in addition to economic impacts strengthens the argument for climate mitigation and or adaptation.

Health outcomes can be directly linked to various environmental determinants already commonly modelled by IAMs and SDMs such as air quality, nutritious food access, water availability, temperature extremes and biodiversity. However, many of these determinants have not yet been linked to health through an analysis or publication by the IAM and SDM community [37].

Additional determinants, as they relate to health outcomes, were mapped out and listed in the supplementary material and can be used as inspiration for future analytical efforts. Synergistic relationships may be found when it comes to the implementation of energy efficient/clean technology and infrastructure. Good infrastructure combats time poverty, enables access to health services, community interaction and employment. Other important components of infrastructure include transportation networks and energy grid connection [48]. End use energy products such as household energy access allow for health-positive changes such as reduced exposure to air pollutants and refrigeration of food. Additionally, as climate-related extreme events and flooding are occurring at an increasing rate, new infrastructure needs to be built to withstand (and rebuild from) these events. However, a large proportion of materials used in construction of the built environment, such as steel and cement are themselves notable emitters CO_2_. Decarbonization and energy efficiency of the building and transportation sector is therefore an important consideration to keep in mind and offers co-benefits for healthier people in urbanized areas and could be another area of investigation [48, 49].

Further, beyond merely survival there are other human needs such as security and self-actualization which, by creating indicators, can represent the possibility for achieving mental and social well-being which may in turn promote more sustainable behaviors in high resource settings. Finally, we hope to argue that through examining actual environmental exposures we may be able capture the synergy between preservation of the environment and human well-being.

## Supplementary Information

**Additional file 1.**

## Data Availability

The dataset used during the current study are available from the corresponding author on reasonable request.
