# Supplementary material for: Healthy lives and well-being for all at all ages: expanding representations of determinants of health within systems dynamics and integrated assessment models
Source: Sustain Earth. 2023 Dec 5;6(1):15. doi: 10.1186/s42055-023-00064-5 (PMC11728693; doi:10.1186/s42055-023-00064-5)
Supplement: Supplementary file 1 — Additional file 1. [file 42055_2023_64_MOESM1_ESM.docx]

Supplementary Information

Environmental Change on Health and Human Well-Being Within Systems Dynamics and Integrated Assessment Models: The Current State

Eartha Weber^1^Msc.; George Downward^2^ PhD.; Maria GM Pinho, PhD^1^.; Detlef P. Van Vuuren^3^ PhD.

^1^ Eartha Weber Copernicus Institute of Sustainable Development, Utrecht University, Utrecht, The Netherlands.

Email: e.d.c.weber@uu.nl (corresponding author).

Maria GM Pinho, PhD, Copernicus Institute of Sustainable Development, Utrecht University, Utrecht, The Netherlands.

^2^ George Downward., Department Population Health Sciences, Julius Center for Health Sciences and Primary Care, Institute for Risk Assessment Sciences, Division of Environmental Epidemiology University Medical Center, Utrecht, The Netherlands (George Downward, PhD)

^3^Detlef van Vuuren,

PBL Netherlands Environmental Assessment Agency, The Hague, The Netherlands.

Copernicus Institute of Sustainable Development, Utrecht University, Utrecht, The Netherlands.

*The authors declare that they have nothing to disclose*

Table 1. Grey Literature Search Documentation

| **Date** | **Organization name; website URL** | **Search strategy(s)/ words searched including (if applicable) how items were selected*.*** | **# items retrieved/ search results** | **# of items screened (uploaded to citation management software)** |
| --- | --- | --- | --- | --- |
| 9-6-2021 | International Futures;  https://pardee.du.edu/ | *Used website*  *Search strategies:*  *Went to Publications tab here they have the following:*   1. *PPHP Series* 2. *Book and Book Chapters* 3. *Journal Articles* 4. *African Futures* 5. *Other PARDEE Publications* 6. *Third Party Publications* 7. *Model Documentation* 8. *Working Papers* | *Search Results:*   1. 5 2. 19 3. 34 4. 27 | *Items Screened:*   1. 1 2. 1 3. 5 4. 3 (specific to Africa) |
| 10-6-2021 | iSDG;  [iSDG - Integrated Sustainable Development Goals Model \| Millennium Institute (millennium-institute.org)](https://www.millennium-institute.org/isdg)  (Threshold 21) | 1. Research Reports 2. Journal Publications | 1. 23 2. 24 | 1. 5 2. 6 |
| 15-6-2021 | International Institute for Applied Systems Analysis (MESSAGEix-GLOBIOM) | *. Used Advanced Search under SHAW (Social Cohesion, Health and Wellbeing or Migration and Sustainable Development ) section* | 1. 12 | 1)0 |
| 15-6-2021 | Cenergia | 1. Recent publications 2. Reports | 1) 41  2) 4 | 1) 1  2) 0 |
| 15-6-2021 | WITCH-GLOBIOM | Search: health   1. Publications | 1) 8 | 1)6 |
| 15-6-2021 | REMIND-MAgPIE  [PIKpublic (pik-potsdam.de)](https://publications.pik-potsdam.de/pubman/faces/SearchResultListPage.jsp?esq=%7B%22bool%22%3A%7B%22must%22%3A%5B%7B%22term%22%3A%7B%22publicState%22%3A%7B%22value%22%3A%22RELEASED%22%2C%22boost%22%3A1.0%7D%7D%7D%2C%7B%22bool%22%3A%7B%22should%22%3A%5B%7B%22simple_query_string%22%3A%7B%22query%22%3A%22health%22%2C%22flags%22%3A-1%2C%22default_operator%22%3A%22and%22%2C%22analyze_wildcard%22%3Atrue%2C%22auto_generate_synonyms_phrase_query%22%3Atrue%2C%22fuzzy_prefix_length%22%3A0%2C%22fuzzy_max_expansions%22%3A50%2C%22fuzzy_transpositions%22%3Atrue%2C%22boost%22%3A1.0%7D%7D%2C%7B%22bool%22%3A%7B%22should%22%3A%5B%7B%22term%22%3A%7B%22objectId%22%3A%7B%22value%22%3A%22health%22%2C%22boost%22%3A1.0%7D%7D%7D%2C%7B%22match%22%3A%7B%22objectPid%22%3A%7B%22query%22%3A%22health%22%2C%22operator%22%3A%22AND%22%2C%22prefix_length%22%3A0%2C%22max_expansions%22%3A50%2C%22fuzzy_transpositions%22%3Atrue%2C%22lenient%22%3Afalse%2C%22zero_terms_query%22%3A%22NONE%22%2C%22auto_generate_synonyms_phrase_query%22%3Atrue%2C%22boost%22%3A1.0%7D%7D%7D%2C%7B%22match%22%3A%7B%22versionPid%22%3A%7B%22query%22%3A%22health%22%2C%22operator%22%3A%22AND%22%2C%22prefix_length%22%3A0%2C%22max_expansions%22%3A50%2C%22fuzzy_transpositions%22%3Atrue%2C%22lenient%22%3Afalse%2C%22zero_terms_query%22%3A%22NONE%22%2C%22auto_generate_synonyms_phrase_query%22%3Atrue%2C%22boost%22%3A1.0%7D%7D%7D%2C%7B%22match%22%3A%7B%22metadata.identifiers.id%22%3A%7B%22query%22%3A%22health%22%2C%22operator%22%3A%22AND%22%2C%22prefix_length%22%3A0%2C%22max_expansions%22%3A50%2C%22fuzzy_transpositions%22%3Atrue%2C%22lenient%22%3Afalse%2C%22zero_terms_query%22%3A%22NONE%22%2C%22auto_generate_synonyms_phrase_query%22%3Atrue%2C%22boost%22%3A1.0%7D%7D%7D%2C%7B%22match%22%3A%7B%22metadata.sources.identifiers.id%22%3A%7B%22query%22%3A%22health%22%2C%22operator%22%3A%22AND%22%2C%22prefix_length%22%3A0%2C%22max_expansions%22%3A50%2C%22fuzzy_transpositions%22%3Atrue%2C%22lenient%22%3Afalse%2C%22zero_terms_query%22%3A%22NONE%22%2C%22auto_generate_synonyms_phrase_query%22%3Atrue%2C%22boost%22%3A1.0%7D%7D%7D%5D%2C%22a) | Search: health   1. Publications | 1. 125 | 1. 28 |
| 15-6-2021 | IMACLIM | publications | 1. 26 | 1. 0 |
| 16-6-2021 | TIAM-UCL |  | 1) 33 | 1) 0 |
| 16-6-2021 | GEM-E3 | publications | 1. 68 | 1. 1 |
| 17-6-2021 | CGIAR research programme libraries IFPRI (IMPACT) model  [Search \| IFPRI : International Food Policy Research Institute](https://www.ifpri.org/list/all/pub/16222/true) | Publications tab (under Impact) | 1. 32 | 1)3 |
| 17-6-2021 | AIM/CGE  <http://www.nies.go.jp/social/en/results.html> | Publications and other Results | Unsure no totals on page | 1. 25 |
| 17-6-2021 | Joint Global Change Research Institute (GCAM) | Search: Health | 1) 312 | 1) 8 |

Table 2. Description and Solution Method of Models

| **Model** | **Purpose** | **Type and Solution Method** |
| --- | --- | --- |
| *Integrated assessment models* | |  |
| BLUES (Brazilian Land Use and Energy System)/Cenergia | Assessment of Brazilian Energy and Land Use system | Optimisation |
| GCAM (Global Change Analysis Model) | Represents the behavior of energy system, water, agriculture and land use, economy and climate. Can be used to explore carbon taxes, carbon trading, regulations and technology deployment. | Hybrid  Simulation |
| IMAGE/GISMO | Quantify changes in Human Development using HDI, population health measures, and MDG indicators. | Hybrid  Simulation |
| REMIND-MAgPIE | Finding the optimal mix of investments in economy and energy sector given population, technology, policy and climate constraints. | Hybrid  Simulation |
| INTERNATIONAL FUTURES^7^ | Integrates 186 countries data to forecast human social and natural systems. In the health model distal drivers like population, income and education are used to determine life expectancy. Stocks are population by age-sex, stunted population and HIV prevalence (meaning that a multiplier is used to determine deaths and subtracted to get the final life expectancy). Flows are births mortality and morbidity. Agent class behavior is also incorporated (smoking, indoor solid fuel use, obesity) | Hybrid  Simulation |
| FeliX (Functional Enviro-economic Linkages Integrated Nexus) | Fast modelling of indicators representing 8 SDGs with a simulation of global scale social, economic and environmental interactions. | Simulation |
| iSDG (Threshold 21) | Policy simulation tool for integrated long term national development planning used to test policy, monitor and evaluate it. | Hybrid  Simulation |
| *Computed General Equilibrium/Partial Equilibrium Models* | | |
| AIM-CGE (Asia- Pacific Integrated Model/Computable General Equilibrium) | Analyses climate change mitigation and its impact. It uses socio-economic scenarios in order to represent energy demand. It generates CO_2_ emissions. | Hybrid  Simulation |
| IMPACT (International Model for Policy Analysis of Agricultural Commodities and Trade) | Analyses water and food security. Represents supply demand and trade, dietary and food preferences of key food commodities. | Simulation and Bottom up- Optimisation |
| Message Globiom | Analyses emission, climate and impacts from energy and land use. Co-links impacts of air pollution and source based aerosol emissions on health as well as household energy consumption. | Optimisation |
| WITCH GLOBIOM | Model linking economy, energy sector, which is lined to Globiom a land use model which covers agriculture and forestry in great detail, and a climate model called MAGICC used to compute future climate impacts which is then linked to TM5-FASST an air pollutant tool. Health impact is measured using a damage function | Bottom-up  Optimisation |

Table 3. References and Data Extracted from Publications by Model

| Model | Distal or Proximal Determinant | Scenarios | Health Related Outcome | Health Analysis Tool | References |
| --- | --- | --- | --- | --- | --- |
| BLUES (Brazilian Land Use and Energy System)/Cenergia | GHG emissions (ENDOGENOUS) which lead to Air Quality measured as a estimate of PM2.5 at a grid level modelled by CALPUFF  Socio-Cultural Drivers (private space -- ideologies surrounding manhood) | Exogenous Energy System Scenarios  Qualitative examination of Brazillian indigenous ideologies and practices | Mortality from Cerebrovascular disease, Chronic Obstructive Pulmonary Disease, Lung Cancer and Lower Respiratory Infection, All-cause infant mortality, Hospital admissions for all cardiovascular, asthma and respiratory conditions  Found that impacts on women and health differ in Brazil due to a different outlook on time and taste preferences. cooking is done outside and deforestation is not done by communities because of their spiritual relationship with trees. | (exogenous) BENMAP-CE  Case-Studies examining behaviourial shift from fuel to LPG | (Howard et al., 2019; Mazzone et al., 2021)(Howard et al., 2019; Mazzone et al., 2021)(Howard et al., 2019; Mazzone et al., 2021)(Howard et al., 2019; Mazzone et al., 2021)(Howard et al., 2019; Mazzone et al., 2021)(Howard et al., 2019; Mazzone et al., 2021) |
| GCAM (Global Change Analysis Model) | GHG emissions which lead to a estimate of Air Quality (PM_2.5_ and O^3^ concentration)  Water Scarcity  Water Contamination  Nighttime Light^a^  Fire Forecasting | RCP and Sectorial ,  Simulations from gasoline and diesel sectors | PM_2.5_ Mortality Costs  PM_2.5_ and Ozone All Cause Deaths  Avoided Premature Mortality  Heat Wave Mortality  Premature Deaths | (exogenous) BENMAP-CE | (Cui et al., 2018; Li et al., 2020; Markandya et al., 2018; Ou et al., 2020; Tang et al., 2022; Wang et al., 2020; Xiong et al., 2022)(Cui et al., 2018; Li et al., 2020; Markandya et al., 2018; Ou et al., 2020; Tang et al., 2022; Wang et al., 2020; Xiong et al., 2022)(Cui et al., 2018; Li et al., 2020; Markandya et al., 2018; Ou et al., 2020; Tang et al., 2022; Wang et al., 2020; Xiong et al., 2022)(Cui et al., 2018; Li et al., 2020; Markandya et al., 2018; Ou et al., 2020; Tang et al., 2022; Wang et al., 2020; Xiong et al., 2022)(Cui et al., 2018; Li et al., 2020; Markandya et al., 2018; Ou et al., 2020; Tang et al., 2022; Wang et al., 2020; Xiong et al., 2022)(Cui et al., 2018; Li et al., 2020; Markandya et al., 2018; Ou et al., 2020; Tang et al., 2022; Wang et al., 2020; Xiong et al., 2022) |
| IMAGE/GISMO | Temperature and Precipitation, Access to Modern Energy Services | SSPs | Malaria Mortality, Diarrhea Mortality, Diarrhea mortality, Nutritional Deficiency Mortality, Pneumonia Mortality, | GISMO | (Dagnachew et al., 2020; Lucas et al., 2019)(Dagnachew et al., 2020; Lucas et al., 2019)(Dagnachew et al., 2020; Lucas et al., 2019)(Dagnachew et al., 2020; Lucas et al., 2019)(Dagnachew et al., 2020; Lucas et al., 2019)(Dagnachew et al., 2020; Lucas et al., 2019) |
| REMIND-MAgPIE | Policies regarding air pollutant concentrations PM_2.5_ , Concentration | SSPs | Human toxicity, DALY attributable to PM-10 | None, indication if concentration is above WHO recommended levels | (Belmin et al., 2021; Budolfson et al., 2021; Creutzig et al., 2022; Luderer et al., 2019)(Belmin et al., 2021; Budolfson et al., 2021; Creutzig et al., 2022; Luderer et al., 2019)(Belmin et al., 2021; Budolfson et al., 2021; Creutzig et al., 2022; Luderer et al., 2019)(Belmin et al., 2021; Budolfson et al., 2021; Creutzig et al., 2022; Luderer et al., 2019)(Belmin et al., 2021; Budolfson et al., 2021; Creutzig et al., 2022; Luderer et al., 2019)(Belmin et al., 2021; Budolfson et al., 2021; Creutzig et al., 2022; Luderer et al., 2019) |
| INTERNATIONAL FUTURES | GDP per capita, Adult Education,Smoking Impact, BMI, Technological Advance | Base Case, IPPC, adaptations to the Rio+ pathways, No-Covid, Current Path, Unequal Paths | Deaths by Cause, Life expectancy (Years of Life Lost, Years of Life Lost to Disability, Disability Adjusted Life Years, Morbidity, Mortality Probability)  Child Stunting, Child Mortality | Integrated into the IF framework | (Birkmann et al., n.d.; Kuhn et al., 2016; Moyer & Bohl, 2019; Technical Assistance Center, n.d.)(Birkmann et al., n.d.; Kuhn et al., 2016; Moyer & Bohl, 2019; Technical Assistance Center, n.d.)(Birkmann et al., n.d.; Kuhn et al., 2016; Moyer & Bohl, 2019; Technical Assistance Center, n.d.)(Birkmann et al., n.d.; Kuhn et al., 2016; Moyer & Bohl, 2019; Technical Assistance Center, n.d.)(Birkmann et al., n.d.; Kuhn et al., 2016; Moyer & Bohl, 2019; Technical Assistance Center, n.d.)(Birkmann et al., n.d.; Kuhn et al., 2016; Moyer & Bohl, 2019; Technical Assistance Center, n.d.) |
| FeliX (Functional Enviro-economic Linkages Integrated Nexus) | GDP | Business as usual, SSP scenarios in combination with RCPs | Life expectancy change as result of GDP | GDP impact on life expectancy | (Moallemi et al., 2022)(Moallemi et al., 2022)(Moallemi et al., 2022)(Moallemi et al., 2022)(Moallemi et al., 2022)(Moallemi et al., 2022) |
| iSDG (Threshold 21) | Health Expenditure as % of GDP | Buisiness as usual, Policy Scenario 1, SDG scenario, National Prospective Study; Base case, Recovered Immunity Protection Variations, Vaccine Protection Variations, Known proportion of cases variations, Lockdown Sensitivity Variations; | None, COVID case fatality, U-5 Mortality Rates | GDP expenditure in healthcare, MDG expenditure | (Armenia et al., 2022; Brereton & Pedercini, 2021; Pedercini et al., 2011, 2018; Pedercini & Barney, 2010)(Armenia et al., 2022; Brereton & Pedercini, 2021; Pedercini et al., 2011, 2018; Pedercini & Barney, 2010)(Armenia et al., 2022; Brereton & Pedercini, 2021; Pedercini et al., 2011, 2018; Pedercini & Barney, 2010)(Armenia et al., 2022; Brereton & Pedercini, 2021; Pedercini et al., 2011, 2018; Pedercini & Barney, 2010)(Armenia et al., 2022; Brereton & Pedercini, 2021; Pedercini et al., 2011, 2018; Pedercini & Barney, 2010)(Armenia et al., 2022; Brereton & Pedercini, 2021; Pedercini et al., 2011, 2018; Pedercini & Barney, 2010) |
| AIM-CGE (Asia- Pacific Integrated Model/Computable General Equilibrium) | Air Quality  Water Scarcity  Water Quality  Heat Exposure Index  Ozone Concentration | SSP1,SSP2, SSP3; BAU, RCP2.6,RCP4.5,RCP8.5, Countermeasure scenario, air policy target scenarios with current leglislation, maximum technically feasible reduction, SWRO (desalination of water feasibility) | Some had none  Avoided premature mortality,  Health expenditure  Childhood Stunting/Underweight  DALY due to stunting  Labour Capacity  DALY/kg-CO_2_  Mortality | Health Assessment Model | (Dong et al., 2015; Hasegawa et al., 2016; Ishida et al., 2014; Kim et al., 2019, 2020; Liu et al., 2020; Phung et al., 2022; Rao et al., 2017; Takakura et al., 2017; Yunus et al., 2020)(Dong et al., 2015; Hasegawa et al., 2016; Ishida et al., 2014; Kim et al., 2019, 2020; Liu et al., 2020; Phung et al., 2022; Rao et al., 2017; Takakura et al., 2017; Yunus et al., 2020)(Dong et al., 2015; Hasegawa et al., 2016; Ishida et al., 2014; Kim et al., 2019, 2020; Liu et al., 2020; Phung et al., 2022; Rao et al., 2017; Takakura et al., 2017; Yunus et al., 2020)(Dong et al., 2015; Hasegawa et al., 2016; Ishida et al., 2014; Kim et al., 2019, 2020; Liu et al., 2020; Phung et al., 2022; Rao et al., 2017; Takakura et al., 2017; Yunus et al., 2020)(Dong et al., 2015; Hasegawa et al., 2016; Ishida et al., 2014; Kim et al., 2019, 2020; Liu et al., 2020; Phung et al., 2022; Rao et al., 2017; Takakura et al., 2017; Yunus et al., 2020)(Dong et al., 2015; Hasegawa et al., 2016; Ishida et al., 2014; Kim et al., 2019, 2020; Liu et al., 2020; Phung et al., 2022; Rao et al., 2017; Takakura et al., 2017; Yunus et al., 2020) |
| IMPACT | nutrition, GHG emissions,  Insufficient Pollination  Taxes on processed meat, socio-economic pathways, | Diet Scenarios (Flexitarian, Pescatarian, Vegetarian, Vegan); No climate Change, Climate Change, Comprehensive investment scenario | PM2.5 Output  DALY due to Chronic and Hidden Hunger  Loss of healthy food consumption and availibility  Attributable deaths | Quantitative foresight modeling | (Smith et al., 2022; Springmann et al., 2016, 2018; Sulser et al., 2021)(Smith et al., 2022; Springmann et al., 2016, 2018; Sulser et al., 2021)(Smith et al., 2022; Springmann et al., 2016, 2018; Sulser et al., 2021)(Smith et al., 2022; Springmann et al., 2016, 2018; Sulser et al., 2021)(Smith et al., 2022; Springmann et al., 2016, 2018; Sulser et al., 2021)(Smith et al., 2022; Springmann et al., 2016, 2018; Sulser et al., 2021) |
| WITCH | Exposure to PM2.5 | Npi scenario,  SSP scenarios | Mortality Rate  Premature Deaths | Damage function | (Reis et al., 2022)(Reis et al., 2022)(Reis et al., 2022)(Reis et al., 2022)(Reis et al., 2022)(Reis et al., 2022) |
| Multiple IAMS in combination with GAINS (Greenhouse gas-Air pollution Interactions and Synergies) model or model inter-comparison projects | Energy Emissions | Reference Scenarios, SSPs | PM2.5 Concentration |  | (Dimitrova et al., 2022; Rafaj et al., 2021; Rao et al., 2016; Xie et al., 2016, 2019)(Dimitrova et al., 2022; Rafaj et al., 2021; Rao et al., 2016; Xie et al., 2016, 2019)(Dimitrova et al., 2022; Rafaj et al., 2021; Rao et al., 2016; Xie et al., 2016, 2019)(Dimitrova et al., 2022; Rafaj et al., 2021; Rao et al., 2016; Xie et al., 2016, 2019)(Dimitrova et al., 2022; Rafaj et al., 2021; Rao et al., 2016; Xie et al., 2016, 2019)(Dimitrova et al., 2022; Rafaj et al., 2021; Rao et al., 2016; Xie et al., 2016, 2019) |
| ^a^ Night time light exposure is potentially associated with impaired cardiometabolic function | | | | | |

Table 4. Plausible Health and Well-Being Impacts Based on Diverse Conceptualizations of Environment

| **Environmental Exposure, Material/Built Environment or Eco-System Service** | **Constituents of Health and Well-Being** | **Known Global Dataset(s)** |
| --- | --- | --- |
| **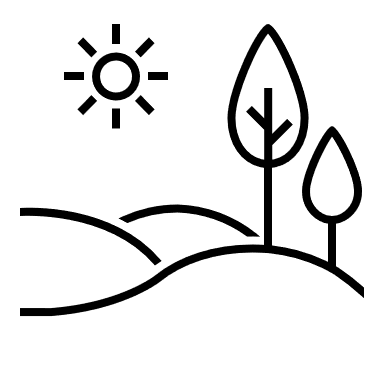**  *Environmental Exposures*   - **Water Pollutants**   - 1. Untreated Water     2. Nitrates in Water     3. Pharmaceuticals/Endocrine Disrupters in Water/PFAs     4. Metals in water - **Food**  1. Access 2. Safety 3. Quality  - **Air Pollutants**  1. Outdoor 2. Indoor  - **Extreme Weather/Events**  1. Temperature 2. Precipitation 3. Floods and Storms 4. Fires  - **Ecosystem Degradation** - **Waste, Chemical or Metal Pollutants** - **Light Pollution** - **Microbiome** | *Physical Illness or Disease*  ***Water-Borne Disease***   1. Infectious Disease (Cryptosporidium, norovirus) 2. Colon Cancer 3. Hormonal Problems/Anti-biotic Resistance/thyroid disease 4. Carcinoma, neuro-behavioral abnormalities, hematological disorders   ***Food Related***   1. Malnutrition Associated Diseases 2. Foodborne Illness (Salmonella, Shigella etc.) 3. Micronutrient deficiencies, metabolic disorders, child development   ***Air Pollutants***   1. Cardiovascular, Respiratory Diseases, Cancers, neurodegenerative Disease 2. U-5 mortality, cancer, respiratory, cardiovascular   **Extreme Weather Events**   1. Heat Stress, cardiovascular, respiratory, vector-borne diseases 2. (indirect – crop yield loss) 3. Injuries, water borne illness 4. (indirect- air pollution)   **Ecosystem Degradation**   1. (indirect- increased mortality from extreme events, food & water quality)   **Waste, Chemical, or Metal Pollutants**   1. Toxicity, cognitive defects, organ damage, (indirect bioaccumulation in food chain 🡪 neurological problems), kidney disease, allergies, pancreatitis, tuberculosis   **Light Pollution**   1. Cardio-metabolic Function`   **Microbiome**   1. Obesity, type 2 diabetes, hepatic steatosis, bowel diseases | **IHME (Global Burden of Disease)**  **UNICEF/WHO Joint Monitoring Programme for Water Supply and Sanitation (JMP)**  **Aqueduct Projected Water Stress**  **FAOSTAT**  **NASA SEDAC Data**  **Harmonized Defense Metorological Satellite Program(DMSP) Nighttime light (NTL)**  **Earth Microbiome Project** |
| - 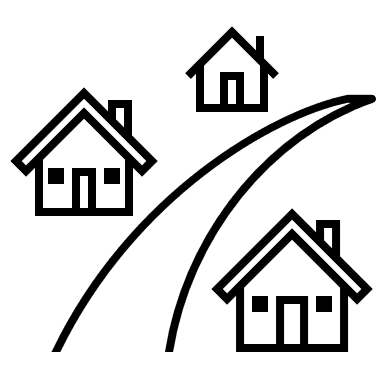*Materials & Built Environment* - Shelter   I)Protection from elements  II)Overcrowdedness/household size   1. Green/Solar roofs  - Energy Access - Heating/Cooling - Refrigeration - Light - Roads & Mobility - Access to services such as Healthcare, Schooling,   - - - Employment - Distance to public transit - Walkability/bikeability - Toilet - Clothing - Communication & Information Access - Public/Community Space - City Planning - Heat Islands | - *Shelter*   i)Violence, Safety/Security, Mental Health, decline in well-being index^46^  ii)Spread of infectious diseases  iii)Urban Heat Island Mitigation   - *Energy Access* - (indirect -reduced indoor air pollution) - Reduced foodborne disease, less undernutrition - Nighttime education and leisure - Roads & Mobility - Emergency healthcare access - Access to markets and services (belonging, self-actualization) - Reduced traffic accidents, increased physical activity - Toilet - Reduction in Diarrhea, norovirus - Clothing - Protection from weather, self-expression - Communication & Information access - Belonging - Public/Community Space - Physical activity, social activity (belonging) - City Planning - Adaptation capacity to extreme temperatures/heat related illness | - **Global Energy Assessment Scenario Database** - **Global Material Flows Database** - **Global Construction Materials Database and Stock Analysis of Residential Buildings between 1970-2050** - **OECD data** - **Facebook Data For Good (Global Electrification Platform, NASA MODIS land cover data, OpenStreetMap)** - **European Space Agency Dataset on cotton** - **Ecoinventory dataset on resources needed to produce Polyester & Fibers** - **Roofpedia** |
| **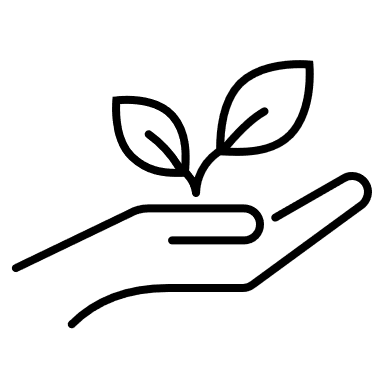**  *Eco-system Services*  *Preserving Nature Provides:*   - Heat Reduction - Stress Reduction - Spaces for Physical and Spiritual Activity - Improved Air Quality - Noise Reduction  1. Traffic noise pollution  - Microbial Diversity - Pollination - Recreation/Tourism   ^47^ | *Heat Reduction*   - Less heat stress, heat related illnesses, nocturnal sleep patterns   *Chronic Stress Results in*   - Memory reduction - Poor lifestyle choices - Stroke and Heart Attacks   *Physical Activity*   - Improved Mood - Reduce Obesity (lack of)   *Noise*   1. Tinnitus, mental health, ischemic heart diseases, sleep disturbance, noise induced hearing loss, metabolic diseases   *Pollination*   - Healthy Food   *Recreation/Tourism*   - Decent work - Physical Activity (dependent on type) | - **Land Use and Land Cover Change** - **Metal Demand for Cars** - **Datasets about average flow of traffic, average speed, road dimenstions** - **European Settlement Map** - **Environmental Noise Directive (END), Copernicus** - **Geostat Grid Dataset** - **GRIP roadtype data** - **OMNO2 Nasa Data** - **Global Multi-Resolution Terrain Elévation Data** |

1. Howard, D. B. *et al.* Health benefits and control costs of tightening particulate matter emissions standards for coal power plants - The case of Northeast Brazil. *Environ Int* **124**, 420–430 (2019).

2. Mazzone, A., Cruz, T. & Bezerra, P. Firewood in the forest: Social practices, culture, and energy transitions in a remote village of the Brazilian Amazon. *Energy Res Soc Sci* **74**, 101980 (2021).

3. Wang, T. *et al.* Health co-benefits of achieving sustainable net-zero greenhouse gas emissions in California. *Nature Sustainability 2020 3:8* **3**, 597–605 (2020).

4. Xiong, Y. *et al.* Long-term trends of impacts of global gasoline and diesel emissions on ambient PM2.5 and O3 pollution and the related health burden for 2000–2015. *Environmental Research Letters* **17**, 104042 (2022).

5. Ou, Y., West, J. J., Smith, S. J., Nolte, C. G. & Loughlin, D. H. Air pollution control strategies directly limiting national health damages in the US. *Nature Communications 2020 11:1* **11**, 1–11 (2020).

6. Cui, R. Y. *et al.* Regional responses to future, demand-driven water scarcity. *Environmental Research Letters* **13**, 094006 (2018).

7. Li, X., Zhou, Y., Zhao, M. & Zhao, X. A harmonized global nighttime light dataset 1992–2018. *Scientific Data 2020 7:1* **7**, 1–9 (2020).

8. Markandya, A. *et al.* Health co-benefits from air pollution and mitigation costs of the Paris Agreement: a modelling study. *Lancet Planet Health* **2**, e126–e133 (2018).

9. Tang, R. *et al.* Air quality and health co-benefits of China’s carbon dioxide emissions peaking before 2030. *Nature Communications 2022 13:1* **13**, 1–9 (2022).

10. Lucas, P. L. *et al.* Future impacts of environmental factors on achieving the SDG target on child mortality—A synergistic assessment. *Global Environmental Change* **57**, (2019).

11. Dagnachew, A. G., Hof, A. F., Lucas, P. L. & van Vuuren, D. P. Scenario analysis for promoting clean cooking in Sub-Saharan Africa: Costs and benefits. *Energy* **192**, 116641 (2020).

12. Belmin, C., Hoffmann, R., Pichler, P. P. & Weisz, H. Fertility transition powered by women’s access to electricity and modern cooking fuels. *Nature Sustainability 2022 5:3* **5**, 245–253 (2021).

13. Luderer, G. *et al.* Environmental co-benefits and adverse side-effects of alternative power sector decarbonization strategies. *Nature Communications 2019 10:1* **10**, 1–13 (2019).

14. Budolfson, M. *et al.* Climate action with revenue recycling has benefits for poverty, inequality and well-being. *Nature Climate Change 2021 11:12* **11**, 1111–1116 (2021).

15. Creutzig, F. *et al.* Demand-side solutions to climate change mitigation consistent with high levels of well-being. *Nat Clim Chang* **12**, 36–46 (2022).

16. Technical Assistance Center, R. THE IMPACT OF COVID-19 ON THE REDUCTION OF CHILD STUNTING OVER THE NEXT TWO DECADES The problem.

17. Birkmann, J. *et al.* Scenarios for vulnerability: opportunities and constraints in the context of climate change and disaster risk. doi:10.1007/s10584-013-0913-2.

18. Kuhn, R., Rothman, D. S., Turner, S., Solórzano, J. & Hughes, B. Beyond Attributable Burden: Estimating the Avoidable Burden of Disease Associated with Household Air Pollution. (2016) doi:10.1371/journal.pone.0149669.

19. Moyer, J. D. & Bohl, D. K. Alternative pathways to human development: Assessing trade-offs and synergies in achieving the Sustainable Development Goals. *Futures* **105**, 199–210 (2019).

20. Moallemi, E. A. *et al.* Early systems change necessary for catalyzing long-term sustainability in a post-2030 agenda. *One Earth* **5**, 792–811 (2022).

21. Pedercini, M., Blanco, S. & Kopainsky, B. Application of the Malaria Management Model to the Analysis of Costs and Benefits of DDT versus Non-DDT Malaria Control. *PLoS One* **6**, e27771 (2011).

22. Pedercini, M. & Barney, G. O. Dynamic analysis of interventions designed to achieve millennium development goals (MDG): The case of Ghana. *Socioecon Plann Sci* **44**, 89–99 (2010).

23. Pedercini, M., Zuellich, G., Dianati, K. & Arquitt, S. Toward achieving Sustainable Development Goals in Ivory Coast: Simulating pathways to sustainable development. *Sustainable Development* **26**, 588–595 (2018).

24. Brereton, C. & Pedercini, M. Covid‐19 case rates in the uk: Modelling uncertainties as lockdown lifts. *Systems* **9**, 60 (2021).

25. Armenia, S., Arquitt, S., Pedercini, M. & Pompei, A. Anticipating human resilience and vulnerability on the path to 2030: What can we learn from COVID-19? *Futures* **139**, (2022).

26. Dong, H. *et al.* Pursuing air pollutant co-benefits of CO2 mitigation in China: A provincial leveled analysis. *Appl Energy* **144**, 165–174 (2015).

27. Ishida, H. *et al.* Global-scale projection and its sensitivity analysis of the health burden attributable to childhood undernutrition under the latest scenario framework for climate change research. *Environmental Research Letters* **9**, 064014 (2014).

28. Kim, S. E. *et al.* Air quality co-benefits from climate mitigation for human health in South Korea. *Environ Int* **136**, 105507 (2020).

29. Yunus, A. P., Masago, Y. & Hijioka, Y. COVID-19 and surface water quality: Improved lake water quality during the lockdown. *Science of The Total Environment* **731**, 139012 (2020).

30. Liu, J. Y. *et al.* The importance of socioeconomic conditions in mitigating climate change impacts and achieving Sustainable Development Goals. *Environmental Research Letters* **16**, 014010 (2020).

31. Phung, V. L. H. *et al.* Environmental variable importance for under-five mortality in Malaysia: A random forest approach. *Science of The Total Environment* **845**, 157312 (2022).

32. Hasegawa, T., Fujimori, S., Takahashi, K., Yokohata, T. & Masui, T. Economic implications of climate change impacts on human health through undernourishment. *Clim Change* **136**, 189–202 (2016).

33. Takakura, J. *et al.* Cost of preventing workplace heat-related illness through worker breaks and the benefit of climate-change mitigation. *Environmental Research Letters* **12**, 064010 (2017).

34. Rao, S. *et al.* Future air pollution in the Shared Socio-economic Pathways. *Global Environmental Change* **42**, 346–358 (2017).

35. Kim, S. E., Hijioka, Y., Nagashima, T. & Kim, H. Particulate Matter and Its Impact on Mortality among Elderly Residents of Seoul, South Korea. *Atmosphere 2020, Vol. 11, Page 18* **11**, 18 (2019).

36. Springmann, M. *et al.* Global and regional health effects of future food production under climate change: a modelling study. *The Lancet* **387**, 1937–1946 (2016).

37. Springmann, M. *et al.* Health-motivated taxes on red and processed meat: A modelling study on optimal tax levels and associated health impacts. *PLoS One* **13**, e0204139 (2018).

38. Smith, M. R. *et al.* The lost opportunity from insufficient pollinators for global food supplies and human health. *Lancet Planet Health* **6**, S3 (2022).

39. Sulser, T. B., Beach, R. H., Wiebe, K. D., Dunston, S. & Fukagawa, N. K. Disability-adjusted life years due to chronic and hidden hunger under food system evolution with climate change and adaptation to 2050. *Am J Clin Nutr* **114**, 550–563 (2021).

40. Reis, L. A., Drouet, L. & Tavoni, M. Internalising health-economic impacts of air pollution into climate policy: a global modelling study. *Lancet Planet Health* **6**, e40–e48 (2022).

41. Dimitrova, A. *et al.* LETTER • OPEN ACCESS Projecting the impact of air pollution on child stunting in India-synergies and trade-offs between climate change mitigation, ambient air quality control, and clean cooking access. *Environ. Res. Lett* **17**, 104004 (2022).

42. Xie, Y., Dai, H., Dong, H., Hanaoka, T. & Masui, T. Economic Impacts from PM 2.5 Pollution-Related Health Effects in China: A Provincial-Level Analysis. (2016) doi:10.1021/acs.est.5b05576.

43. Rafaj, P. *et al.* Air quality and health implications of 1.5 °C–2 °C climate pathways under considerations of ageing population: a multi-model scenario analysis. *Environmental Research Letters* **16**, 045005 (2021).

44. Xie, Y. *et al.* Comparison of health and economic impacts of PM2.5 and ozone pollution in China. *Environ Int* **130**, 104881 (2019).

45. Rao, S. *et al.* A multi-model assessment of the co-benefits of climate mitigation for global air quality. *Environmental Research Letters* **11**, 124013 (2016).

46. Precarious Housing and Wellbeing - Shelter WA. https://www.shelterwa.org.au/precarious-housing-and-wellbeing-a-multi-dimensional-investigation/.

47. 2 Ecosystems and Their Services.
